# Impact of stent graft geometry on hemodynamics in superior mesenteric artery reconstruction after fenestrated/branched endovascular aneurysm repair

**DOI:** 10.3389/fbioe.2026.1901373

**Published:** 2026-09-15

**Authors:** ShuTing Gao, TianMing Huang, MengQiang Hu, BingJing Chen, XiaoYu Qi, YuanMing Luo, YiQing Li, Chao Yang

**Affiliations:** 1 Department of Vascular Surgery, Union Hospital, Tongji Medical College, Huazhong University of Science and Technology, Wuhan, China; 2 Department of Technology, Boea Wisdom (Hangzhou) Network Technology Co., Ltd., Hangzhou, China; 3 Department of Mechanical Engineering, The University of Iowa, Iowa, IA, United States

**Keywords:** fenestrated/branched endovascular aneurysm repair, geometric configuration, hemodynamic, parametric modeling, SMA stent grafts

## Abstract

**Background:**

During visceral artery reconstruction in fenestrated/branched endovascular aneurysm repair (F/B-EVAR), the superior mesenteric artery (SMA) plays a critical role in intestinal perfusion and often exhibits complex postoperative geometry. In this study, we employed computational fluid dynamics (CFD) with virtual modeling to simulate various SMA reconstruction strategies and systematically evaluate the impact of postoperative geometric configurations on SMA hemodynamics.

**Methods:**

Virtual parametric models representing 12 postoperative geometric configurations were artificially reconstructed from CTA images. Proximal parameters of the superior mesenteric artery stent, including the stent graft inflow angle (θ) and intraluminal length-to-aortic diameter ratio (L/D), as well as the distal parameter, the outlet angle (α), were defined to differentiate these 12 configurations. Hemodynamic parameters Pressure ratio (the SMA outlet pressure/descending aortic inlet pressure), flow fraction (branch flow/total aortic inflow), and hemodynamic indices, including time-averaged wall shear stress and oscillatory shear index were defined to evaluate hemodynamic differences across the configurations.

**Results:**

PR decreased up to 5.2% for maximum as θ increased. Retrograde configurations (θ > 90°) generated inlet vortices during deceleration, most pronounced at θ = 180°. Variation in L/D did not produce a meaningful change in PR, whereas increasing L/D was associated with a more pronounced local flow disturbance beneath the protruding segment. Vortical structures formed beneath stent segments protruding into the aorta during systole, with vortex size positively correlated with L/D and more pronounced during systolic deceleration. PR values were similar among models with different α configurations. But hemodynamic indices indicated that a larger outlet angle α conferred greater benefits to the endothelial environment. The patient-specific illustrations showed consistency with these findings.

**Conclusion:**

This study elucidates the hemodynamic consequences of varying SMA stent graft configurations during F/B-EVAR and provides biomechanical insights to aid optimal clinical decision-making for SMA stent graft positioning.

## 1 Introduction

Fenestrated/branched endovascular aneurysm repair (F/B-EVAR) has become an important treatment option for complex thoracoabdominal aortic aneurysms (Huang et al., 2018). By preserving perfusion to vital visceral branches while minimizing operative trauma compared with open repair (Jongkind et al., 2010), F/B-EVAR substantially decreases perioperative morbidity and mortality (Huang et al., 2018; Chiu and Chien, 2011; EVAR trial participants, 2004; Lederle et al., 2009; Greenhalgh et al., 2010). However, challenges, including relatively high endoleak rates and uncertain long-term durability, remain (Gallitto et al., 2022). The long-term patency of visceral artery branches remains a key predictor of clinical outcomes since branch occlusion or stenosis is directly associated with organ ischemia, higher reintervention rates, and even mortality (Meuli et al., 2025). The superior mesenteric artery (SMA) plays a critical role in intestinal perfusion and often exhibits complex postoperative geometry. Due to its unique structural configuration, which resembles that of the aorta and includes an arched section, the fluid dynamics are comparatively complex. The incidence of bowel ischemia after F/B-EVAR is around 1%–2.7% (Gallitto et al., 2021; Farber et al., 2017; Westin et al., 2020), which is influenced by the geometric configuration of the SMA stent graft (Wang et al., 2025). However, there are no standardized guidelines regarding the specific landing details of SMA stent grafts in F/B-EVAR. This is particularly evident in the use of physician-modified endografts (PMEGs), where such decisions rely heavily on operator experience (Gao and Guo, 2022; Tsilimparis et al., 2024). The long-term adverse effects of suboptimal stent geometry, such as excessive protrusion or retrograde placement, are a major concern for inner branches. However, how the geometric configurations of SMA stent grafts affect hemodynamics, which is associated with branch-vessel patency, remains unclear.

Conventional imaging modalities, such as CTA, provide detailed anatomical information but cannot assess the functional hemodynamic condition. Computational fluid dynamics (CFD) overcomes these limitations by simulating flow conditions and provides an insightful tool to quantify detailed hemodynamic metrics (Kalipcilar and Salman, 2023; Ong et al., 2020; Lu et al., 2016; Malatos et al., 2024). The CFD method was employed to evaluate the optimal configuration of SMA stent placement in the endovascular treatment of superior mesenteric artery dissection (SMAD) (Mei et al., 2025), and to explore the hemodynamic implications of renal artery reconstruction during F/B-EVAR (Wang et al., 2025; Kandail et al., 2015). Given the critical role of SMA in intestinal perfusion and its unique anatomical characteristics, the hemodynamic study focusing on the SMA in F/B-EVAR remains limited.

In this study, we developed parametric models with designed proximal and distal geometric configurations of the SMA stent graft. Our goal is to investigate the hemodynamic difference induced by the varying configurations of the SMA stent graft, which may be beneficial for the improvement of surgical strategies and long-term SMA patency after F/B-EVAR.

## 2 Materials and methods

### 2.1 Materials

We retrospectively reviewed patients undergoing F/B-EVAR at our institution between January 2021 and December 2024. Inclusion criteria included SMA endovascular reconstruction, CTA images eligible for model reconstruction used in the CFD analysis, and at least 12-month postoperative follow-up. The clinical endpoints included SMA-related adverse outcomes: (1) stent stenosis/occlusion, (2) endoleaks (Type I-III), (3) mesenteric ischemic events, (4) all-cause mortality, and (5) target vessel reintervention. The study consisted of a primary parameter-controlled CFD analysis and a subsequent patient-specific illustration analysis. Two base models of eligible cases with two common anatomical configurations of the SMA, differing in SMA-aorta angle and arch curvature (one featured a larger SMA-aorta angle and a pronounced arch, whereas the other showed the opposite pattern), were selected to design virtual parametric models. Then postoperative cases matching parametric configurations were included to illustrate whether the hemodynamic patterns observed in parametric simulations could be observed in real anatomical scenarios. These patient-specific models were not included in parameter-controlled comparisons and were not intended as formal validation cohorts. All procedures were performed by the same experienced operator (C.Y.). Medtronic (Minneapolis, MN, United States of America) or Lifetech Scientific (Shenzhen, China) was used for the main body, and Gore Viabahn (W. L. Gore and Associates, Flagstaff, AZ, United States of America) was used for bridging and inner branches reconstruction of the SMA.

Imaging was acquired with a third-generation dual-source CT scanner (SOMATOM Force, Siemens AG, Erlangen, Germany) at 100 kV, 250 mAs, 0.25 s rotation time, 250 mm field of view (FOV), and 0.5 mm slice thickness, with an in-plane resolution of 0.5 mm. CTA datasets were first used to reconstruct patient-specific aortic geometries. To minimize inter-observer variability, each dataset was independently reconstructed by two experienced biomedical engineers using the commercially available software DetecModeling (v1.0, Boea Wisdom, Hangzhou, China) (Zhang et al., 2026; Liu and Cui, 2026; Yang et al., 2026), supervised by a radiologist. A third senior technician determined the final models used in CFD simulation. The workflow included segmentation, 3D reconstruction, and controlled smoothing. The reconstructed geometry extended from the descending aorta to the iliac arteries and included the proximal segments of visceral branches.

### 2.2 Virtual parametric model designing

Three primary geometric parameters of the SMA stent graft were defined as shown in Figure 1a. The length-to-diameter (L/D) ratio is defined as the ratio of the SMA stent intraluminal length to the aortic diameter at the same ostium. The inflow angle (θ) defined the inflow direction of SMA flow: θ < 90° represents an antegrade flow from the aorta into the stent; θ > 90° represents a retrograde situation. In addition, the outlet angle (α) denotes the outflow angle from the distal end of the SMA stent. Based on α, distal landing situations are classified as: obtuse angle configuration (α > 90°), right angle configuration (α = 90°), and acute angle configuration (α < 90°). Combining the defined geometric parameters, three groups of parametric models were generated based on the two selected base models by varying proximal and distal SMA stent-graft configurations through deformation, resection, and geometric adjustments. To isolate the independent effect of each parameter on flow behavior, a single parameter was altered per group while the remaining parameters were kept unchanged. The graft and stent structures were not explicitly modeled in the present CFD analysis. Instead, the reconstructed postoperative vessel lumen was used as the fluid domain, assuming that the stented graft surfaces were fully endothelialized. Thus, the simulations focused on the hemodynamic consequences of the reconstructed vascular geometry and stent configuration rather than direct flow interaction with the graft or stent material.

**FIGURE 1:**
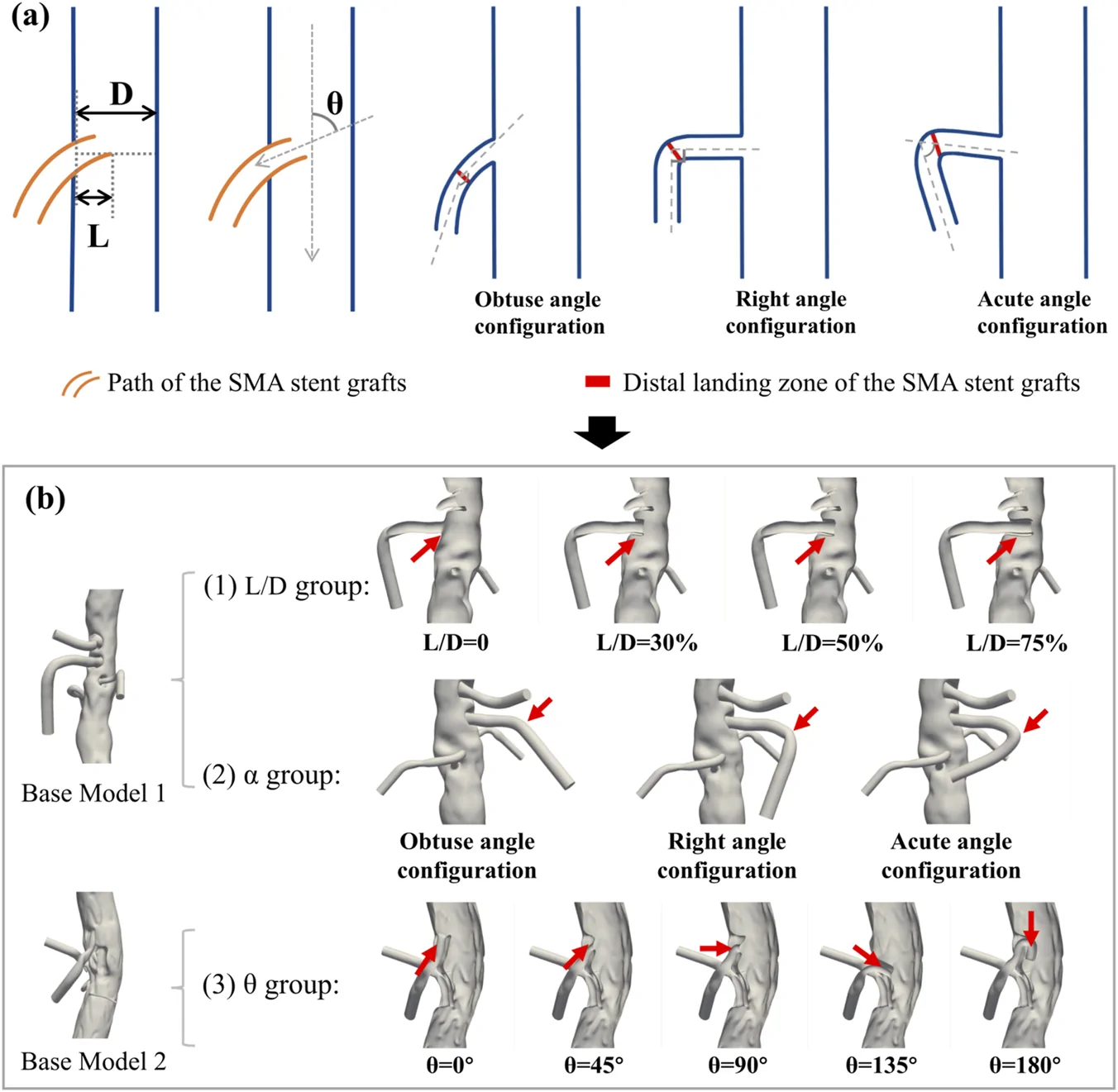
Definition of geometric parameters for the SMA stent graft and model construction. **(a)** Schematic illustration of the geometric parameters used to characterize superior mesenteric artery (SMA) stent graft configurations. *L* denotes the maximum protrusion length of the stent into the aortic lumen, measured on a plane perpendicular to the aortic centerline, while *D* represents the aortic diameter on the same plane. The length-to-diameter (L/D) ratio is defined to quantify the degree of stent protrusion into the aorta. *θ* indicates the angle between the SMA inflow direction and the aortic flow axis. *α* refers to the angulation between the stent and the distal vessel segment. Based on α, distal landing types are classified as obtuse angle configuration (α > 90°), right angle configuration (α = 90°), and acute angle configuration (α < 90°). **(b)** Parametric models were constructed for computational simulations based on two base models: the L/D group, α group, and θ group, each systematically varying a single geometric parameter while keeping the others constant. The arrows indicate the locations of the variables.

### 2.3 Hemodynamic simulation and analysis

All models were meshed using a tetrahedral grid with boundary-layer refinement (five prismatic layers) in DetecFluid (Boea Wisdom, Hangzhou, China) (Liu and Cui, 2026; Yang et al., 2026; Liu et al., 2025) to accurately capture near-wall hemodynamics. The appropriate mesh size was determined through a grid independence check. Although grid-independence testing was performed, no dedicated numerical uncertainty quantification was conducted in the present study. Therefore, very small differences in global hemodynamic parameters were interpreted cautiously and were not used alone to support quantitative hemodynamic conclusions. An empirical waveform with a flat velocity profile was applied at the descending aorta inlet (Les et al., 2010). Boundary conditions were kept identical in each parametric group. A three-element Windkessel model was applied to all outlets, with proximal resistance, distal resistance, and compliance estimated following established parameter-fitting methods to reproduce physiologic systolic and diastolic pressures (Liu et al., 2025).

Blood was assumed to be an incompressible Newtonian fluid (density 1,060 kg/m^3^, viscosity 0.0035 Pa s), with a rigid vessel wall under no-slip conditions. Individual stent struts and graft structures were not explicitly resolved in the CFD model. Instead, the reconstructed postoperative lumen was treated as the fluid domain, with a continuous, fully endothelialized luminal surface assumed. The SST k-ω model was employed within a Reynolds-averaged Navier-Stokes (RANS) framework for the transient simulations, given its established performance in complex aortic hemodynamics and its ability to robustly handle flow separation and adverse-pressure-gradient conditions (Cheng et al., 2014; Stokes et al., 2023a; Stokes et al., 2023b). The use of the RANS formulation was not intended to imply that the flow was fully turbulent throughout the vascular domain; rather, the same turbulence-modeling framework was applied consistently across all parametric configurations to facilitate comparative assessment of their relative hemodynamic effects. Spatial discretization used a second-order upwind scheme; temporal integration used a first-order backward Euler scheme. The PISO algorithm was employed for pressure-velocity coupling (Moukalled, 2016). Convergence tolerance for all variables was set at 10^−4^. Each simulation ran for three cardiac cycles (1000-time steps per cycle), and the results of the final cycle were used in the analysis to ensure temporal convergence.

In this study, focus was emphasized on the flow distribution, pressure characteristics, and local flow patterns within the SMA segment. Key hemodynamic parameters were calculated, including time-averaged wall shear stress (TAWSS) and oscillatory shear index (OSI).

TAWSS measures the time-averaged friction between blood flow and the vessel wall, and is calculated as

$$\text{TAWSS}=\frac{1}{T}\int_{0}^{T}\left|\tau_{w}\right|dt$$

The vector **τ**_**w**_ is the wall shear stress (WSS) vector, and *T* is the cardiac cycle.

OSI is the directional variation of the WSS vector, with values ranging from 0 to 0.5, and is calculated as

$$\text{OSI}=\frac{1}{2}\left(1-\frac{\left|\int_{0}^{T}\tau_{w}dt\right|}{\int_{0}^{T}\left|\tau_{w}\right|dt}\right)$$

Referring to the concept of fractional flow reserve (FFR), pressure was adopted as a critical hemodynamic metric in this study. Organ perfusion is primarily determined by the pressure gradient between the SMA and the aorta. The metric pressure ratio (PR = P_1_/P_0_) was defined, where P_0_ represents the descending aortic inlet pressure and P_1_ the SMA outlet pressure, serving as an index of SMA flow reserve. PR values closer to one indicate lower resistance and better perfusion. Flow fraction (SMA flow divided by the total aortic inflow) was included as a supplementary metric.

### 2.4 Patient-specific models for illustration

Patient-specific models of patients who met the inclusion criteria and matched the discussed parametric configurations were established to illustrate the hemodynamic results of parametric models. To ensure comparability of the selected cases, only cases with complete endovascular revascularization of all four visceral arteries (celiac artery, SMA, and bilateral renal arteries) were included. Because the proximal and distal configurations of SMA stent grafts exhibit substantial inter-patient variability, we selected cases based on the θ, L/D, and α, enabling the analysis of a single configuration under consistent values of the remaining parameters. Additionally, cases with minimal celiac artery protrusion length upstream were selected to reduce upstream flow-interaction effects. All procedures, including postoperative image acquisition, geometric reconstruction, image processing, hemodynamic simulation and analysis, as well as the software and the personnel involved, were uniform across all patients.

## 3 Results

### 3.1 Modeling results

Three groups of parametric models based on the two selected base models were presented in Figure 1b. Based on the standard anatomical configuration (base model 1), two parametric groups were constructed: (1) L/D varied group, L/D varied among 0, 30%, 50%, and 75%, and kept θ = 90° and right angle configuration (α = 90°) constant; (2) α-varied group, three parametric models were established (obtuse, right, and acute angle configuration), with fixed L/D (30%) and θ (90°). In F/B-EVAR procedures, variations in the SMA inflow angle are primarily associated with the use of inner branches. Given that base model two allows for a greater range of variation in inflow angle compared with base model 1, the θ-varied group was constructed based on base model 2. Specifically, five parametric models (θ = 0°, 45°, 90°, 135°, and 180°) were constructed with identical L/D (30%) and α (right angle configuration). The selected parameter ranges covered a spectrum of postoperative geometric configurations, including unfavorable boundary conditions, to explore their hemodynamic implications rather than to define clinical recommendations.

### 3.2 Effect of the θ on SMA hemodynamics

PR values decreased to 0.9142, 0.9092, 0.9033, 0.8776, and 0.8669 respectively during the inflow angle θ increased to 0°, 45°, 90°, 135°, and 180° (Figure 2a). Differences were modest (<5%), but retrograde angles (θ > 90°) were associated with relatively lower PR. The flow fraction (Figure 2b) and pressure contours (Figure 2c) also showed similar trends. At peak systole, flow remained organized across all models (Figure 2d). During systolic deceleration, inlet vortices formed notably in the two retrograde configurations (135° and 180°), especially at θ = 180°.

**FIGURE 2:**
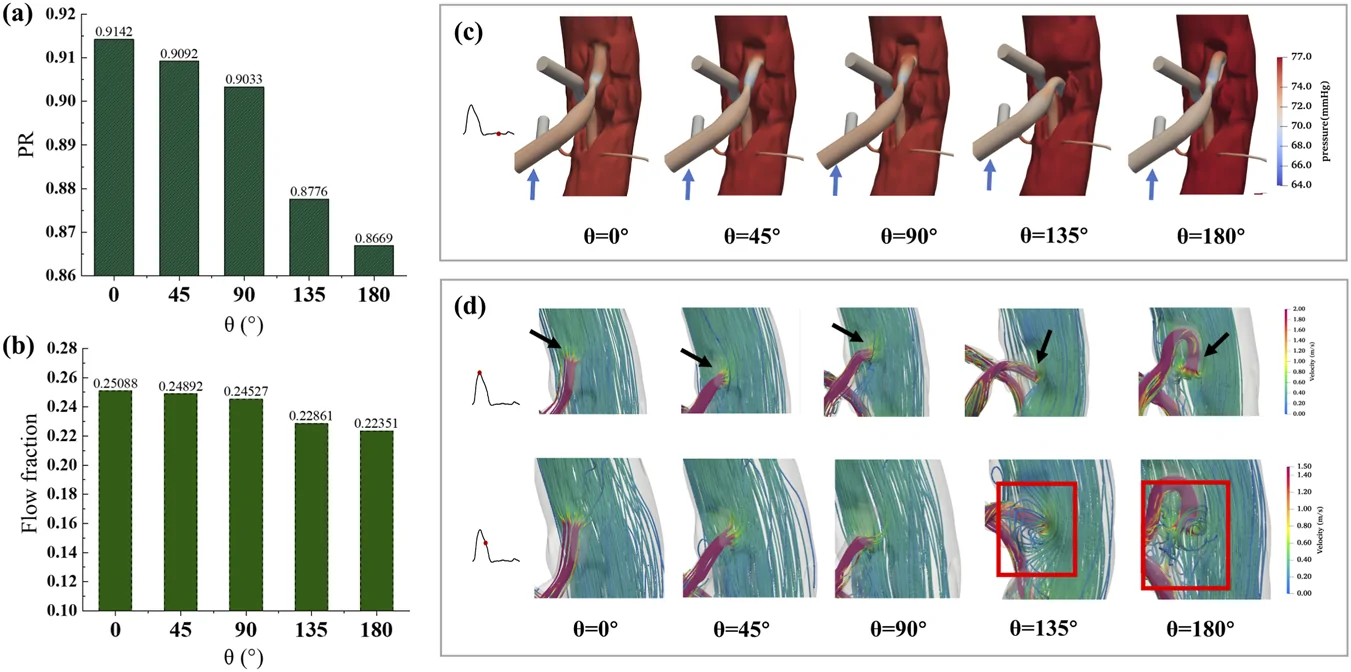
Hemodynamic assessment of the θ group. **(a)** Pressure ratio (PR); **(b)** flow fraction; **(c)** pressure contours; and **(d)** velocity streamline patterns for models with varying inflow angle (θ). Increasing θ was associated with a progressive decline in PR and flow fraction, while pressure contour distributions showed a consistent trend. Blue arrows indicate the superior mesenteric artery (SMA), and the black arrows denote the stent inlet. Notably, retrograde configurations (θ > 90°) generated distinct inlet vortices (boxes) during the deceleration phase of systole, which became most pronounced at θ = 180°. The apparent narrowing in **(C)** represents the transition zone between the bridging stent graft and native vessel and was reconstructed based on postoperative CTA morphology.

### 3.3 Effect of the L/D ratio on SMA hemodynamics

As the L/D ratio increased from 0% to 75%, PR remained highly similar across configurations, with values of 0.9721, 0.9699, 0.9695, and 0.9639, respectively (Figure 3a). The maximum absolute difference was 0.0082 (0.82%). Given the absence of dedicated numerical uncertainty quantification and the small magnitude of this difference, it was not interpreted as a meaningful quantitative change in SMA perfusion. In contrast, increasing L/D was associated with progressively more pronounced vortical flow structures beneath the protruding segment, particularly during systolic deceleration (Figure 3d). Mid-to-late diastolic pressure contours (Figure 3c) showed similar distribution patterns among models, while SMA flow fractions also remained consistent across L/D values (Figure 3b).

**FIGURE 3:**
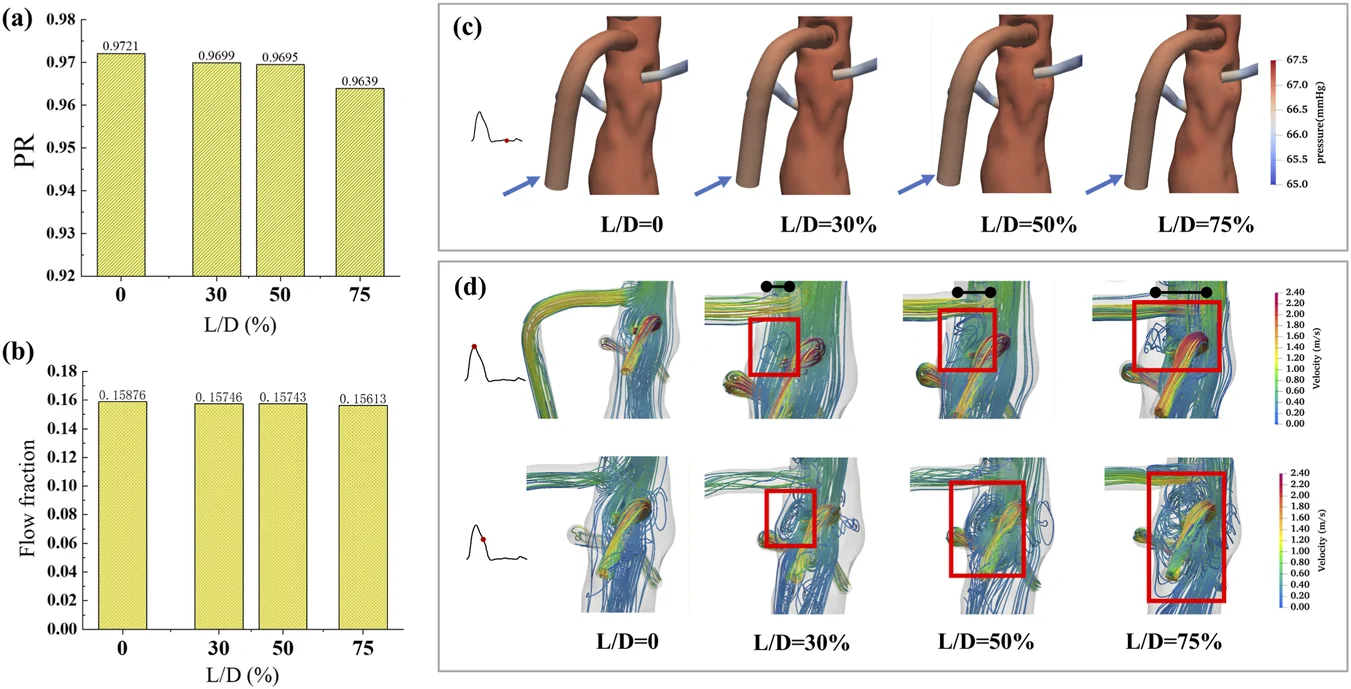
Hemodynamic assessment of the L/D group. **(a)** Pressure ratio (PR); **(b)** flow fraction; **(c)** pressure contours; and **(d)** velocity streamline patterns for models with varying length-to-diameter (L/D) ratios. The PR values were similar among the different L/D configurations, with a maximum absolute difference of 0.0082 (0.82%), and this small difference was not interpreted as a meaningful quantitative change in SMA perfusion. Similarly, flow-fraction and pressure-contour distributions remained consistent. Blue arrows indicate the superior mesenteric artery (SMA). Black line segments denote the extent of SMA stent protrusion into the aortic lumen. Notably, vortical flow structures (boxes) were observed beneath stent segments protruding into the aorta, becoming more prominent with increasing L/D ratios and particularly accentuated during systolic deceleration.

### 3.4 Effect of the α on SMA hemodynamics

PR values were 0.9683, 0.9699, and 0.9641 for the obtuse, right, and acute outlet-angle configurations, respectively, with a maximum absolute difference of 0.0058 (0.58%) (Figure 4a). The SMA flow fractions also remained consistent (Figure 4b). Given the small magnitude of this difference, the variation in PR was not interpreted as a meaningful quantitative effect of α on SMA perfusion. In contrast, outlet angle produced more apparent differences in local flow organization. The obtuse-angle configuration showed a relatively smooth flow path, whereas the right- and acute-angle configurations introduced progressively more pronounced directional changes. These geometric alterations may modify the local wall-shear environment and potentially influence endothelial mechanobiological responses (Chiu and Chien, 2011). Therefore, TAWSS and OSI were further evaluated to characterize the local hemodynamic effects of different outlet-angle configurations (Figure 4c).

**FIGURE 4:**
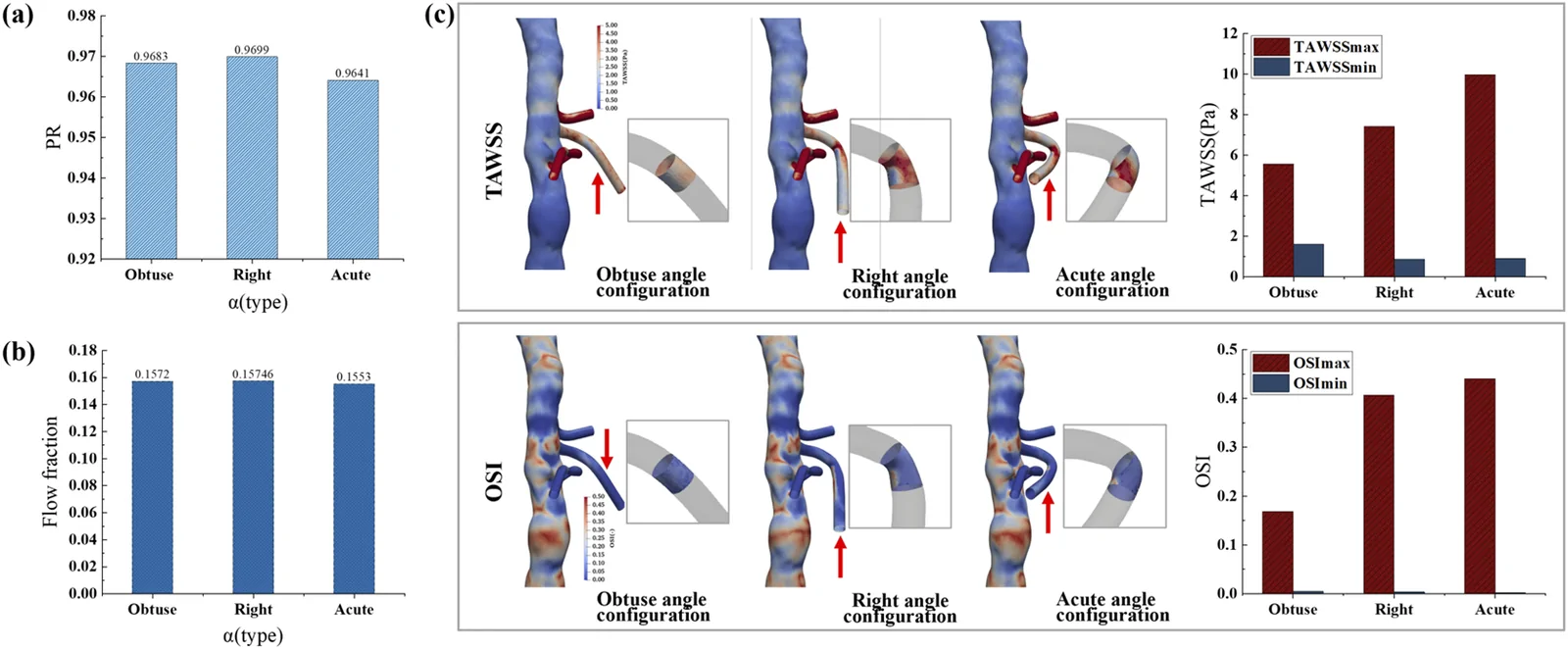
Hemodynamic and wall shear stress–related parameters of the α group. **(a)** Pressure ratio (PR); **(b)** flow fraction; and **(c)** distributions of time-averaged wall shear stress (TAWSS) and oscillatory shear index (OSI), along with bar charts of the corresponding maximum and minimum values for each parameter. PR and flow fraction showed relatively limited variation among α groups. Arrows indicate the superior mesenteric artery (SMA). In contrast, variations in outlet angle (α) were associated with differences in local hemodynamic characteristics, as reflected by differences in TAWSS and OSI distributions, suggesting differences in local wall shear environments.

### 3.5 Distribution characteristics of hemodynamic parameters

TAWSS contours showed that, in the obtuse angle configuration, SMA TAWSS distribution was relatively uniform, with only a mildly reduced area on the aortic side near the SMA bend. Right and acute angle configurations exhibited pronounced high-shear regions at the post-bend impact zone and low-shear regions on the aortic side. Maximum TAWSS increased progressively as α decreased: obtuse angle configuration: 5.5591 Pa, right angle configuration: 7.4136 Pa, acute angle configuration: 9.9587 Pa. The minimum TAWSS was highest in the obtuse angle configuration (1.6087 Pa), while the other two were lower and similar (right angle configuration: 0.8654 Pa, acute angle configuration: 0.9047 Pa).

OSI contours showed that OSI distribution was relatively uniform in the obtuse angle configuration, while the right and acute angle configurations exhibited localized areas of increased OSI on the aortic side after the bend. As the α decreased, the maximum OSI gradually increased (obtuse angle configuration: 0.1687, right angle configuration: 0.4068, acute angle configuration: 0.4403), with the latter approaching 0.5, indicating strong flow reversal in that area; the minimum OSI values were similar among the three models. Thus, the influence of α was more apparent in local wall-shear-related patterns than in global SMA perfusion.

### 3.6 Results of patient-specific models for illustration

To assess the clinical feasibility of the findings obtained from the parametric models, six eligible cases with geometric configurations matching the parametric groups were chosen for comparison (Table 1). No SMA-related adverse events occurred in any of the cases during follow-up. Patients 1–3 were selected for the effects of L/D. Their inflow angles (θ = 45°–50°) were similar, while L/D values were 32%, 45%, and 75%, respectively, with all three demonstrating obtuse angle configuration. Analysis of postoperative SMA pressure ratios and flow fractions (Table 1) revealed no consistent correlation with L/D. Streamline visualization (Figure 5) showed relatively uniform flow distribution within the SMA at peak systole across all cases. As L/D increased, the extent of aortic flow disturbance downstream of the protruding segment increased. These findings were consistent with the parametric-model results.

**TABLE 1:** Baseline hemodynamic parameters, procedural characteristics, and postoperative SMA-related metrics in six cases.

Patient	1	2	3	4	5	6
Systolic pressure (mmHg)	125	126	130	121	126	154
Diastolic pressure (mmHg)	62	60	80	72	74	65
Heart rate (bpm)	83	65	78	76	99	82
Celiac artery						
Been reconstructed	Yes	Yes	Yes	Yes	Yes	Yes
L/D (%)	22	0	0	18	20	43
SMA						
L/D (%)	32	45	75	50	50	50
θ (°)	45	47	50	55	77	77
α (type)	Obtuse	Obtuse	Obtuse	Obtuse	Obtuse	Right
PR	0.9157	0.9474	0.9205	0.9283	0.8922	0.8709
Flow fraction (%)	15.1	11.2	12.0	11.2	11.1	11.1

L/D = length-to-diameter ratio; θ = inflow angle; α = outlet angle types; PR, pressure ratio. Patients 1–3 were used to evaluate the effect of L/D; patients 2, 4, and five were used to assess the impact of θ; in addition, patients five and six were selected to illustrate the influence of α.

**FIGURE 5:**
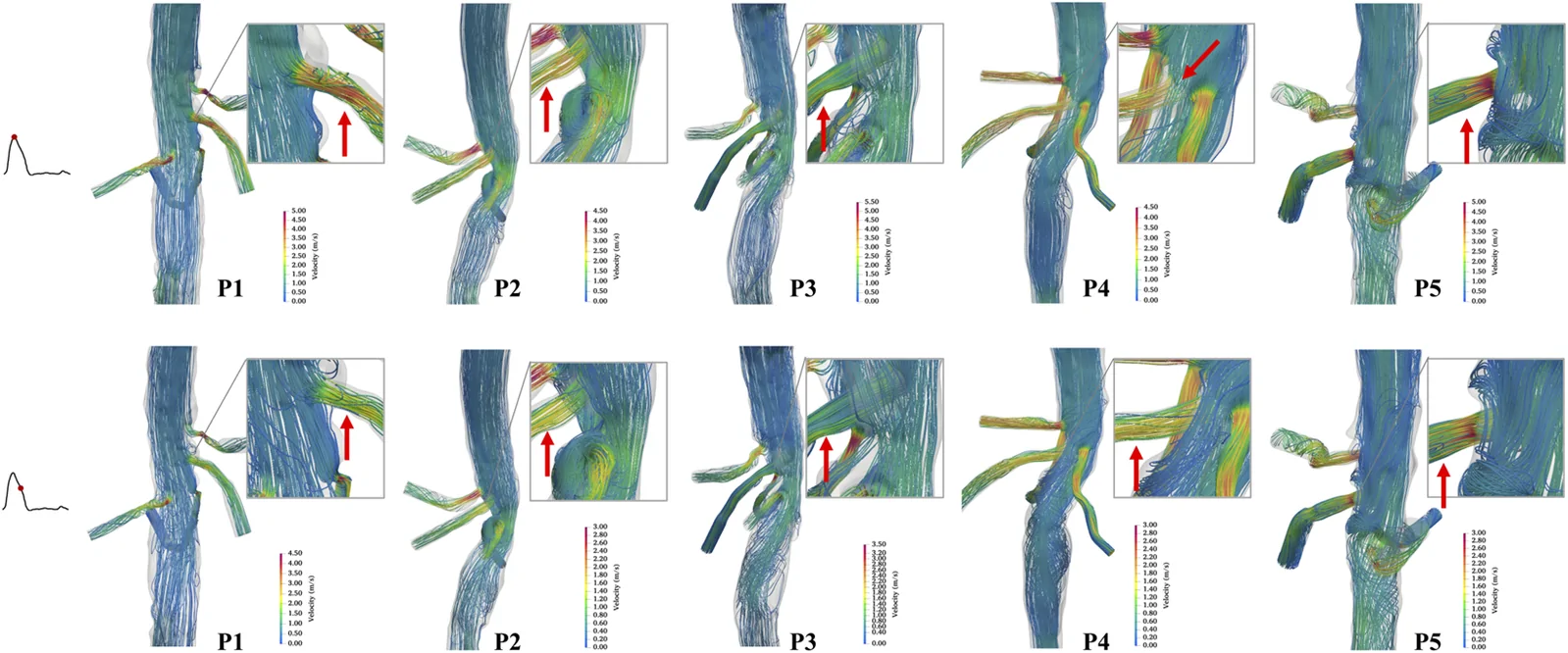
Postoperative velocity streamline patterns in the aortic visceral artery segment of patients 1–5. Velocity streamline patterns are presented for five representative postoperative patients (P1–P5). Arrows indicate the superior mesenteric artery (SMA). Patients 1–3 (P1–P3) were selected to assess the effects of the L/D ratio. As L/D increased, a greater extent of flow disturbance was observed in the aortic lumen downstream of the stent protrusion segment. Patients 2 (P2), 4 (P4), and 5 (P5) were further analyzed to assess the impact of the inflow angle (θ). In these cases (θ < 90°), no evident vortical structures were observed at the stent inlet during systolic deceleration or early diastole. These patient-specific findings were in good agreement with the parametric simulation results.

Patients 2, 4, and five were selected for θ effects. Their L/D values were comparable (∼50%), all were obtuse angle configurations, and their θ angles were 47°, 55°, and 77°, respectively. SMA pressure ratios showed a decreasing trend with increasing θ, while flow fractions showed no consistent pattern (Table 1). Streamline analysis (Figure 5) demonstrated undisturbed aortic flow at peak systole and regular or helical flow within the SMA. No vortical structures were observed at the stent inlet during systolic deceleration or early diastole when θ < 90°. These findings were consistent with the parametric results. No clinical cases with θ > 90° (retrograde configuration) were available, so this condition could not be directly assessed.

Only one patient with a right angle configuration was available, while all other cases showed an obtuse angle configuration. No acute angle configuration cases were identified. Therefore, patients five (obtuse angle configuration) and 6 (right angle configuration) were compared. Given the larger difference in celiac artery L/D (20% vs. 43%), direct comparison of SMA pressure ratios was avoided. Distal SMA hemodynamic indices were analyzed: For patient 6 (right angle configuration), TAWSSmax (7.38633 Pa) was lower than in patient 5 (21.2418 Pa); OSImax was markedly elevated (0.481178 vs. 0.368072). Figure 6 shows the distribution of TAWSS and OSI in the SMA postoperatively for the two cases. Patient five showed more uniform WSS distribution, while patient six exhibited prominent oscillatory and stagnation-prone regions at the post-bend SMA segment. These results were consistent with the trend observed in parametric models.

**FIGURE 6:**
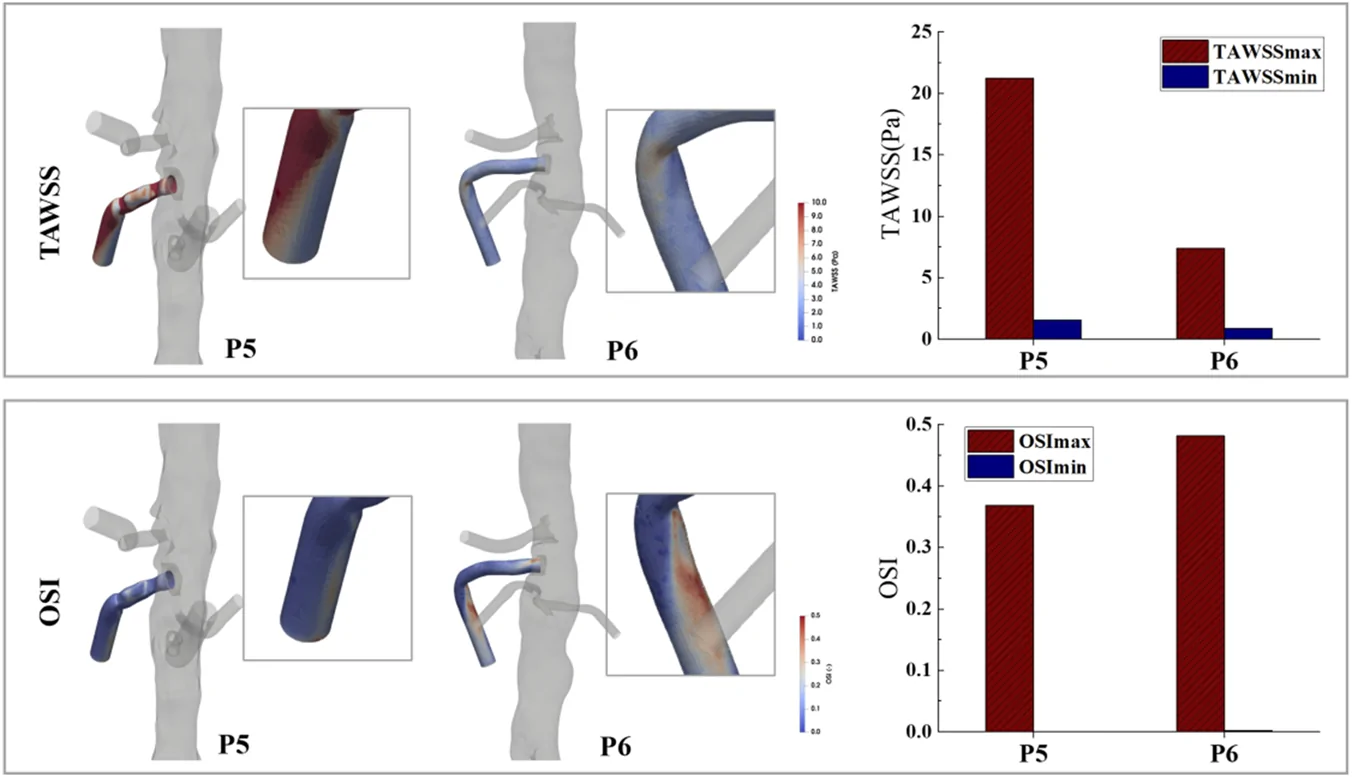
Distribution of TAWSS and OSI in the SMA segment and comparison of maximum and minimum values in post-operative analysis for patient five and patient 6. The distribution of time-averaged wall shear stress (TAWSS) and oscillatory shear index (OSI) within the superior mesenteric artery (SMA) segment is shown for two representative patients, P5 and P6, following stent graft implantation. Patient 5 (P5) is classified as an obtuse angle configuration, and patient 6 (P6) as a right angle configuration. Bar charts represent the maximum and minimum values of TAWSS and OSI for each parameter in the post-operative phase. The hemodynamic parameters at the stent terminus for both patients showed consistent results with those observed in the corresponding model configurations.

## 4 Discussion

In this study, we analyzed the configurations of SMA stent grafts, specifically the inflow angle (θ), the intraluminal length-to-aortic diameter ratio (L/D), and the outflow angle (α), which is closely related to the operative details that surgeons should focus on. Among these, a favorable inflow and outflow angle and an appropriate L/D ratio appear to be the primary determinants of a favorable flow environment. Antegrade configurations (θ < 90°) were associated with more favorable SMA hemodynamic characteristics and smoother inlet flow, while retrograde configurations showed more pronounced inlet vortex formation. While protrusion length had a limited effect on SMA flow-related parameters, excessively high L/D ratios may increase the likelihood of vortex formation and alert the local aortic hemodynamic environment. Lastly, larger α configurations were associated with more uniform distribution of TAWSS, and lower OSI, suggesting relatively favorable local hemodynamic characteristics in obtuse angle configurations. These findings demonstrate that SMA reconstruction is not merely a technical step, but also involves geometric considerations that may have potential implications for hemodynamic behavior and, possibly, longer-term outcomes. However, the relatively small differences in PR associated with L/D (0.82%) and α (0.58%) should be interpreted cautiously. Although grid-independence testing was performed, dedicated numerical uncertainty quantification was not conducted in the present study. Therefore, these sub-1% differences were not considered sufficiently robust to support a quantitative interpretation of changes in SMA perfusion. Accordingly, the interpretation of L/D and α focused primarily on the more apparent changes in local flow structures and wall-shear-related parameters rather than these small differences in global PR. Meanwhile, although reduced branch perfusion pressure or flow may theoretically contribute to organ hypoperfusion, no validated cutoff values currently exist for PR or flow fraction to define clinically relevant SMA ischemia after F/B-EVAR. Therefore, the differences observed in this study should be interpreted as relative hemodynamic variations between geometric configurations rather than direct indicators of ischemic risk.

The inflow angle (θ) reflects how well the stent aligns with the main aortic flow. In clinical practice, the choice of θ is generally determined by aortic anatomy and the access. In complex cases, retrograde configuration deployment may be necessary. Increasing θ, particularly beyond 90°, forces blood to undergo an abrupt directional change, which inevitably leads to flow separation, recirculation, and energy dissipation (Milnor, 1972), resulting in perfusion of branches. This phenomenon has been extensively described in studies on carotid bifurcation flow (Zalud et al., 2023; Bantwal et al., 2021) and aortic arch branch reconstruction with various stent types (Che et al., 2023; Lu et al., 2023). When θ is small (antegrade configuration), the axial momentum of aortic flow can be effectively redirected into the SMA, minimizing energy loss. Kandail et al. also found that retrograde branches lead to minimum renal flow compared to antegrade branches through hemodynamic analysis of the reconstruction of renal artery branches in F/B-EVAR (Kandail et al., 2015). Regretfully, in clinical cases of this study, all SMA stents are deployed in an antegrade orientation so that retrograde configurations could not be assessed. Overall, the closer the stent aligns with the native direction of blood flow, the more physiologically favorable the reconstruction is likely to be. Thus, during F/B-EVAR planning, smaller inflow angles were associated with improved hemodynamic performance. An antegrade, wall-apposed inner-branch configuration (sutured to the main graft, θ ≈ 0°) may be preferable when technically feasible.

In contrast to θ, L/D had little apparent influence on global SMA perfusion in the present simulations, which is consistent with findings from a study on chimney stent grafts of varying lengths utilized in renal arteries (Brand et al., 2024). This may be explained by the fact that branch flow is primarily governed by pressure gradient and inflow direction, rather than the degree of geometric protrusion into the aorta. This aligns with classical hemodynamic theory (Milnor, 1972), where side-branch flow is relatively insensitive to moderate upstream disturbances unless these changes significantly alter the pressure distribution that drives perfusion.

However, increasing L/D introduces a greater extent of stent protrusion into the aortic lumen, which perturbs the main flow and was associated with shear layer separation and more pronounced unsteady swirling flow downstream of the protruding segment. Similar phenomena have been described in a hemodynamic study on renal artery anatomy treated in F/B-EVAR (Wang et al., 2025), where excessive protrusion leads to a larger vortex region and increased branch return flow rate. Excessive protrusion may also increase fatigue-related risks for compliant stents; however, the potential implication requires further biomechanical investigation. Overall, surgeons have relatively greater flexibility in selecting the intraluminal protrusion length of the stent, however, excessive protrusion may induce additional flow disturbances and should be considered with caution.

The outflow angle (α) appears to have a predominantly localized effect, primarily influencing flow behavior at the distal segment of the stent. A smaller α creates a sharper transition between the stent and the native distal vessel, which was associated with more concentrated flow impingement on the vessel wall and subsequent regions of flow deceleration and recirculation. This combination of flow acceleration and subsequent stagnation promotes an uneven distribution of wall shear forces, typically characterized by reduced TAWSS and increased OSI in the impacted regions. Such hemodynamic conditions have been associated with endothelial dysfunction and adverse vascular remodeling (Chiu and Chien, 2011). In particular, regions of low TAWSS combined with high OSI are known to promote a pro-inflammatory endothelial phenotype, impair nitric oxide production, and facilitate leukocyte adhesion, which may contribute to processes involved in intimal hyperplasia and adverse vascular remodeling. These associations have been consistently demonstrated across a wide range of vascular studies, including carotid bifurcation (Bantwal et al., 2021; Ku et al., 1985), coronary arteries (Wang et al., 2023), and bypass grafts (Pahlavanian and Ganji, 2023), and are widely accepted as key mechanistic pathways underlying vascular complications. Therefore, although the overall impact of α may be less pronounced than that of inflow geometry, acute outlet angle was associated with less favorable local hemodynamic patterns in the present simulations and may warrant careful consideration during procedural planning.

Given the physiological bend of SMA, post-operative outlet configuration is primarily influenced by the distal landing location. Landing proximal to the bend typically results in an obtuse angle configuration, whereas landing at the bend, especially with stents of lower compliance, predisposes to right or acute angle configurations. Therefore, placement of the distal landing zone proximal to the physiological bend may be considered, as this configuration demonstrated more favorable local hemodynamic characteristics in the present simulations. When compliant devices are used, and major branches (e.g., the middle colic artery) are avoided, distal extension beyond the bend may also be acceptable. Overall, operators should make case-specific decisions based on individual anatomy and device performance, aiming for a larger α configuration to minimize adverse hemodynamic environments.

By systematically evaluating geometric parameters of the SMA stent graft in F/B-EVAR, our study provides a more structured framework for understanding how reconstruction geometry may influence SMA durability. Rather than viewing these parameters independently, they should be considered as an integrated system that defines the overall configuration of the stent-graft. Previous studies (Kandail et al., 2015; Brand et al., 2024; Wang et al., 2025) have primarily focused on branch vessel alignment, chimney stent configurations, or renal artery reconstruction. In contrast, the present study extends this field by investigating the influence of distal landing zone geometry on SMA reconstruction after F/B-EVAR. In general, for the reconstruction of secondary aortic branch vessels, regardless of whether fenestration, branched stent grafts, chimney, or periscope techniques are used, maintaining favorable alignment between the inflow direction of the branch and the physiological aortic flow axis may help optimize local hemodynamic conditions. Meanwhile, excessive intraluminal protrusion should be carefully considered. Regarding the selection of the distal landing zone, relatively less tortuous vessel segments may be beneficial for maintaining a straighter postoperative geometry, which in turn could benefit the endothelial environment.

This study has several limitations that should be acknowledged. First, the clinical feasibility assessment cohort was relatively small and did not cover all investigated geometric configurations, which may limit the generalizability of the findings. Larger, multicenter, and prospective studies are warranted to confirm the robustness and validity of these results across diverse anatomical and procedural settings. Second, the retrospective nature of the study introduces inherent risks of selection bias and unmeasured confounding, despite efforts to standardize data collection and analysis. In addition, the duration of follow-up was relatively limited. Notably, no SMA-related adverse events were observed during follow-up, precluding meaningful case–control analyses for clinical endpoints such as target vessel occlusion or reintervention. Future studies, although adverse flow patterns have been associated with vascular remodeling in previous studies, the present CFD analysis cannot establish a direct relationship between hemodynamic parameters and long-term target vessel outcomes. Meanwhile, the findings of the present study should be interpreted within the assumptions and parameter space of the current computational framework. Given the limited range of anatomical and physiological conditions investigated, these results may not be directly generalizable to all SMA reconstruction scenarios and should therefore be considered as hemodynamic insights. Longer-term follow-up is needed to determine the durability of these findings and their relevance to long-term patency.

## 5 Conclusion

CFD provides detailed and quantitative mechanistic insights into how the postoperative configuration of the SMA influences local blood flow patterns in F/B-EVAR. In this parametric study, an antegrade configuration (θ < 90°) was associated with more favorable global SMA perfusion, while limited protrusion was associated with less pronounced local flow disturbance. Outlet angle (α) appears to be an important geometric factor affecting local wall-shear characteristics, with an obtuse-angle configuration (α > 90°) producing relatively favorable local hemodynamic patterns. These findings provide biomechanical insights for SMA reconstruction planning, while further clinical studies are required to determine their implications for target vessel outcomes.

## Data Availability

The original contributions presented in the study are included in the article/supplementary material, further inquiries can be directed to the corresponding authors.

## References

[B1] Bantwal A. Singh A. Menon A. R. Kumar N. (2021). Hemodynamic study of blood flow in the carotid artery with a focus on carotid sinus using fluid-structure interaction. J. Fluids Eng. 144, 021403. 10.1115/1.4051902

[B2] Brand M. Ben-Gur H. Halak M. (2024). The effect of the length of chimney's protrusion on the hemodynamics of abdominal aorta stent graft after endovascular aneurysm repair. Med. Eng. Phys. 134, 104256. 10.1016/j.medengphy.2024.104256 39672659

[B3] Che Y. Zhao J. Zhang X. Luo M. Cao X. Zheng L. (2023). Hemodynamics of different configurations of the left subclavian artery parallel stent graft for thoracic endovascular aortic repair. Comput. Methods Programs Biomed. 241, 107741. 10.1016/j.cmpb.2023.107741 37544164

[B4] Cheng Z. Juli C. Wood N. B. Gibbs R. G. J. Xu X. Y. (2014). Predicting flow in aortic dissection: comparison of computational model with PC-MRI velocity measurements. Med. Eng. Phys. 36, 1176–1184. 10.1016/j.medengphy.2014.07.006 25070022

[B5] Chiu J.-J. Chien S. (2011). Effects of disturbed flow on vascular endothelium: pathophysiological basis and clinical perspectives. Physiol. Rev. 91, 327–387. 10.1152/physrev.00047.2009 21248169 PMC3844671

[B6] Farber M. A. Eagleton M. J. Mastracci T. M. Mckinsey J. F. Vallabhaneni R. Sonesson B. (2017). Results from multiple prospective single-center clinical trials of the off-the-shelf p-Branch fenestrated stent graft. J. Vasc. Surg. 66, 982–990. 10.1016/j.jvs.2017.01.068 28559176

[B7] Gallitto E. Faggioli G. Melissano G. Fargion A. Isernia G. Lenti M. (2021). Preoperative and postoperative predictors of clinical outcome of fenestrated and branched endovascular repair for complex abdominal and thoracoabdominal aortic aneurysms in an Italian multicenter registry. J. Vasc. Surg. 74, 1795–1806.e6. 10.1016/j.jvs.2021.04.072 34098004

[B8] Gallitto E. Faggioli G. Melissano G. Fargion A. Isernia G. Bertoglio L. (2022). Fenestrated and branched endografts for post-dissection thoraco-abdominal aneurysms: results of a national multicentre study and literature review. Eur. J. Vasc. Endovasc. Surg. 64, 630–638. 10.1016/j.ejvs.2022.06.019 35764243

[B9] Gao J. Guo W. (2022). Present and perspective of fenestrated and branched endovascular repair for complex abdominal aortic aneurysm. Chin. J. Vasular Surg. 2, 82–88.

[B10] Greenhalgh R. M. Brown L. C. Kwong G. P. S. Powell J. T. Thompson S. G. , and EVAR trial participants (2004). Comparison of endovascular aneurysm repair with open repair in patients with abdominal aortic aneurysm (EVAR trial 1), 30-day operative mortality results: randomised controlled trial. Lancet 364, 843–848. 10.1016/S0140-6736(04)16979-1 15351191

[B11] Greenhalgh R. M. Brown L. C. Powell J. T. Thompson S. G. Epstein D. Sculpher M. J. (2010). Endovascular *versus* open repair of abdominal aortic aneurysm. N. Engl. J. Med. 362, 1863–1871. 20382983 10.1056/NEJMoa0909305

[B12] Huang I. K. H. Renani S. A. Morgan R. A. (2018). Complications and reinterventions after fenestrated and branched EVAR in patients with paravisceral and thoracoabdominal aneurysms. Cardiovasc Interv. Radiol. 41, 985–997. 10.1007/s00270-018-1917-0 29511866

[B13] Jongkind V. Yeung K. K. Akkersdijk G. J. M. Heidsieck D. Reitsma J. B. Tangelder G. J. (2010). Juxtarenal aortic aneurysm repair. J. Vasc. Surg. 52, 760–767. 10.1016/j.jvs.2010.01.049 20382492

[B14] Kalipcilar A. Salman H. E. (2023). Hemodynamic effects of intraluminal thrombus burden in an idealized abdominal aortic aneurysm. J. Braz. Soc. Mech. Sci. Eng. 45, 508. 10.1007/s40430-023-04441-1

[B15] Kandail H. Hamady M. Xu X. Y. (2015). Comparison of blood flow in branched and fenestrated stent-grafts for endovascular repair of abdominal aortic aneurysms. J. Endovasc. Ther. 22, 578–590. 10.1177/1526602815587261 25979146

[B16] Ku D. N. Giddens D. P. Zarins C. K. Glagov S. (1985). Pulsatile flow and atherosclerosis in the human carotid bifurcation. Positive correlation between plaque location and low oscillating shear stress. Arteriosclerosis 5, 293–302. 10.1161/01.atv.5.3.293 3994585

[B17] Lederle F. A. Freischlag J. A. Kyriakides T. C. Padberg F. T. JR. Matsumura J. S. Kohler T. R. (2009). Outcomes following endovascular vs open repair of abdominal aortic aneurysm: a randomized trial. JAMA 302, 1535–1542. 10.1001/jama.2009.1426 19826022

[B18] Les A. S. Yeung J. J. Schultz G. M. Herfkens R. J. Dalman R. L. Taylor C. A. (2010). Supraceliac and infrarenal aortic flow in patients with abdominal aortic aneurysms: mean flows, waveforms, and allometric scaling relationships. Cardiovasc Eng. Technol. 1. (1), 39–51. 10.1007/s13239-010-0004-8 24324530 PMC3855240

[B19] Liu W. Cui C. (2026). Computational fluid dynamics evaluation of physician-modified stent graft repair for distal residual stanford type B aortic dissection. BMC Cardiovasc Disord. 10.1186/s12872-026-06172-2 42399855 PMC13602504

[B20] Liu Y. Li W. Ding Z. Tang Z. Luo Y. Hu J. (2025). Long-term longitudinal computational study of a Marfan syndrome patient after hybrid repair of aortic arch dissection with parallel stent-grafts. Int. J. Numer. Method Biomed. Eng. 41, e70018. 10.1002/cnm.70018 39979238

[B21] Lu Y.-H. Mani K. Panigrahi B. Hsu W.-T. Chen C.-Y. (2016). Endoleak assessment using computational fluid dynamics and image processing methods in stented abdominal aortic aneurysm models. Comput. Math. Methods Med. 2016, 9567294. 10.1155/2016/9567294 27660648 PMC5021907

[B22] Lu K. Qin W. Sun X. Si Y. Ding G. Fu W. (2023). Computational study of fenestration and parallel grafts used in TEVAR of aortic arch aneurysms. Int. J. Numer. Method Biomed. Eng. 39, e3664. 10.1002/cnm.3664 36447341

[B23] Malatos S. Fazzini L. Raptis A. Nana P. Kouvelos G. Tasso P. (2024). Evaluation of hemodynamic properties after chimney and fenestrated endovascular aneurysm repair. Ann. Vasc. Surg. 104, 237–247. 10.1016/j.avsg.2023.12.084 38492732

[B24] Mei J. Yan H. Zhao X. Yuan Y. Su H. Xue T. (2025). In-stent restenosis after stenting for superior mesenteric artery dissection is associated with stent landing zone: from clinical prediction to hemodynamic mechanisms. J. Endovasc. Ther. 32, 2169–2182. 10.1177/15266028241241494 38561992

[B25] Meuli L. Kaufmann Y. L. Lattmann T. Attigah N. Dick F. Mujagic E. (2025). Editor's choice - peri-operative mortality and morbidity of complex abdominal aortic aneurysm repair in Switzerland: a swissvasc report. Eur. J. Vasc. Endovasc. Surg. 69, 25–35. 10.1016/j.ejvs.2024.06.022 38906370

[B26] Milnor W. R. (1972). Pulsatile blood flow. N. Engl. J. Med. 287, 27–34. 10.1056/nejm197207062870108 4555147

[B27] Moukalled F. M. L. A. (2016). The Finite Volume Method in Computational Fluid Dynamics. Springer International Publishing.

[B28] Ong C. W. Wee I. Syn N. Ng S. Leo H. L. Richards A. M. (2020). Computational fluid dynamics modeling of hemodynamic parameters in the human diseased aorta: a systematic review. Ann. Vasc. Surg. 63, 336–381. 10.1016/j.avsg.2019.04.032 31344467

[B29] Pahlavanian M. H. Ganji D. D. (2023). Computational assessment of venous anastomosis angles and graft configurations in arteriovenous graft. Results Eng. 17, 100944. 10.1016/j.rineng.2023.100944

[B30] Stokes C. Ahmed D. Lind N. Haupt F. Becker D. Hamilton J. (2023a). Aneurysmal growth in type-B aortic dissection: assessing the impact of patient-specific inlet conditions on key haemodynamic indices. J. R. Soc. Interface 20, 20230281. 10.1098/rsif.2023.0281 37727072 PMC10509589

[B31] Stokes C. Haupt F. Becker D. Muthurangu V. VON Tengg-Kobligk H. Balabani S. (2023b). The influence of minor aortic branches in patient-specific flow simulations of Type-B aortic dissection. Ann. Biomed. Eng. 51, 1627–1644. 10.1007/s10439-023-03175-4 36967447 PMC10264290

[B32] Tsilimparis N. Gouveia E Melo R. Tenorio E. R. Scali S. Mendes B. Han S. (2024). Multicenter study on physician-modified endografts for thoracoabdominal and complex abdominal aortic aneurysm repair. Circulation 150, 1327–1342. 10.1161/CIRCULATIONAHA.123.068587 38989565

[B33] Wang X. Shen Y. Shang M. Liu X. Munn L. L. (2023). Endothelial mechanobiology in atherosclerosis. Cardiovasc Res. 119, 1656–1675. 10.1093/cvr/cvad076 37163659 PMC10325702

[B34] Wang Y. Sang Y. Li W. Zhou M. Zhao Y. He X. (2025). A computational study on renal artery anatomy in patients treated with fenestrated or branched endovascular aneurysm repair. Bioeng. (Basel) 12, 482. 10.3390/bioengineering12050482 40428101 PMC12109362

[B35] Westin G. G. Rockman C. B. Sadek M. Ramkhelawon B. Cambria M. R. Silvestro M. (2020). Increased ischemic complications in fenestrated and branched endovascular abdominal aortic repair compared with standard endovascular aortic repair. J. Vasc. Surg. 72, 36–43. 10.1016/j.jvs.2019.09.044 32081484

[B36] Yang C. Shao S. Leng X. Qi W. Chen Y. Huang L. (2026). Computational fluid dynamics in predicting renal malperfusion after aortic dissection repair. JACC Case Rep. 31, 106060. 10.1016/j.jaccas.2025.106060 41247329 PMC12926182

[B37] Zalud N. C. Bulusu K. V. Plesniak M. W. (2023). Shear stress metrics associated with pro-atherogenic high-risk anatomical features in a carotid artery bifurcation model. Clin. Biomech. (Bristol) 105, 105956. 10.1016/j.clinbiomech.2023.105956 37098301

[B38] Zhang X. Yang M. Huang T. Qin Q. Qian P. Luo Y. (2026). Assessment of cardiac allograft vasculopathy in heart transplant patients using multidimensional dynamic CTA and principal components analysis. BMC Med. Imaging 26, 294. 10.1186/s12880-026-02368-2 42050431 PMC13267395

